# Factors Associated with the Detection of Actionable Genomic Alterations Using Liquid Biopsy in Biliary Tract Cancer

**DOI:** 10.3390/cancers17183071

**Published:** 2025-09-19

**Authors:** Hiroshi Shimizu, Rei Suzuki, Hiroyuki Asama, Kentaro Sato, Kento Osawa, Rei Ohira, Keisuke Kudo, Mitsuru Sugimoto, Hiromasa Ohira

**Affiliations:** Department of Gastroenterology, Fukushima Medical University School of Medicine, Fukushima 960-1295, Japan; smizu23@fmu.ac.jp (H.S.); asamax@fmu.ac.jp (H.A.); s-ken@fmu.ac.jp (K.S.); osawa@fmu.ac.jp (K.O.); reiohira@fmu.ac.jp (R.O.); h-5361@fmu.ac.jp (K.K.); kita335@fmu.ac.jp (M.S.); h-ohira@fmu.ac.jp (H.O.)

**Keywords:** biliary tract cancer, cholangiocarcinoma, genomic profile, liquid biopsy

## Abstract

**Background**: Blood-based comprehensive genomic profiling (CGP), a form of liquid biopsy, is often used for biliary tract cancer (BTC) when tissue-based CGP (tissue CGP) is unavailable, despite lower detection rates. This study explored factors linked to detecting actionable genomic alterations to optimize its use. **Methods**: We retrospectively analyzed BTC cases in Japan’s C-CAT (June 2019–January 2025), restricting panel comparisons to FoundationOne^®^ CDx (F1; n = 5019) and FoundationOne^®^ Liquid CDx (F1L; n = 1550). Missing covariates were handled by multiple imputations (m = 20). Between-panel balance used 1:1 propensity-score matching (caliper 0.2). Outcomes were modeled with logistic regression. Targets included MSI-H, TMB-H, *FGFR2*/*RET*/*NTRK* fusions, *BRAF* V600E, *KRAS* G12C, *IDH1* mutations, and *ERBB2* amplification. An exploratory analysis stratified results by the number of prespecified enrichment factors (0–4). Liquid biopsy was performed using plasma-based comprehensive genomic profiling assays (FoundationOne^®^ Liquid). **Results**: Missingness was low; after matching (n = 1549 per group) covariates were well balanced (all|SMD|≤0.05). Detection of any actionable alteration was lower with F1L than F1 (16.8% vs. 24.8%; OR 0.61, 95% CI 0.49–0.75; *p* < 0.001). F1L also had lower TMB-H (OR 0.62, 0.43–0.90; *p* = 0.01) and *ERBB2* amplification (OR 0.42, 0.31–0.57; *p* < 0.001), with no significant differences for MSI-H, *IDH1*, *KRAS* G12C, or *BRAF* V600E. Within F1L, non-perihilar location (OR 2.05), liver (1.90), lymph-node (1.41), and lung metastases (1.52) predicted detection of actionable genomic alterations. F1L detection increased from 5.8% (zero factors) to 32.8% (four factors), approximating tissue at three factors. **Conclusions**: The utility of liquid biopsy can be maximized by carefully selecting samples on the basis of conditions that increase the detection rate.

## Simple Summary

Liquid biopsy is often used for biliary tract cancer (BTC) when tissue testing is not possible, but it usually finds fewer targetable (actionable) genomic changes. Using Japan’s national CGP database (C-CAT), we compared matched patients tested with a tissue panel (F1) or a blood panel (F1L). Overall, liquid biopsy detected fewer actionable alterations than tissue testing (16.8% vs. 24.8%). Within the liquid-biopsy group, four readily available clinical features—non-perihilar (non-pCCA) tumor location and the presence of liver, lymph-node, or lung metastases—were each linked to higher detection. The chance of finding an actionable alteration rose stepwise from 5.8% when none of these factors were present to 32.8% when all four were present and approached tissue-testing rates when three factors were present. These results show that choosing liquid biopsy for patients with these features can substantially improve its yield and help clinicians decide when blood-based testing is most likely to be informative.

## 1. Introduction

Biliary tract cancer (BTC), which includes intrahepatic, perihilar, and distal cholangiocarcinoma, as well as gallbladder cancer, is a rare but aggressive malignancy with a poor prognosis [1]. The global incidence of BTC varies geographically, with higher rates observed in Southeast Asia and parts of South America. It is often associated with certain risk factors, such as primary sclerosing cholangitis, liver fluke infection, and chronic biliary inflammation [2]. Early-stage BTC is typically asymptomatic, leading to delayed diagnosis at advanced stages. Surgical resection offers the only potential cure for BTC; however, the majority of patients are diagnosed with unresectable disease. For advanced BTC, systemic chemotherapy, which commonly involves immune checkpoint inhibitors combined with gemcitabine and cisplatin, is the standard first-line treatment [3,4,5].

According to the NCCN guidelines, comprehensive genomic profiling (CGP) is recommended for patients with unresectable or metastatic BTC who are candidates for systemic therapy [6]. CGP conducted using a next-generation sequencing (NGS) panel that includes clinically relevant targets may improve the likelihood of identifying actionable genomic alterations. Notably, druggable aberrations, such as *FGFR2* fusions (9–15% in intrahepatic cholangiocarcinoma [iCCA]), *IDH1* mutations (10–20%), *HER2* overexpression or amplification (5–20% in cholangiocarcinoma [CCA] and 15–30% in gallbladder cancer [GBCa]), are observed with variable frequencies across biliary tract cancers. These alterations are clinically relevant because they correspond to specific therapeutic targets: FGFR inhibitors target aberrant FGFR signaling, *IDH1* inhibitors act on mutant *IDH1* enzyme activity, and HER2-targeted therapies inhibit HER2-mediated signaling pathways [7,8,9]. In cases where tumor tissue is limited or unavailable, repeat biopsy should be considered on the basis of tumor accessibility and patient safety. Alternatively, when tumor tissue is inadequate or unobtainable for NGS, plasma circulating tumor DNA (ctDNA) analysis for CGP, a form of liquid biopsy in cancer genomics, can be considered. In biliary tract disease, liquid biopsy not only facilitates detection of actionable genomic alterations but also shows promise for differentiating malignant from benign entities and for prognostic assessment, as suggested by recent studies [10,11].

This liquid biopsy, however, has intrinsic analytic constraints—most notably its dependence on circulating tumor fraction (ctF). Copy-number gains (e.g., *ERBB2* amplification) and certain structural variants generally require higher ctF for reliable detection, and plasma-based tumor mutational burden (bTMB) can be biased downward when few tumor molecules are captured. Pre-analytical variables and clonal hematopoiesis may further complicate interpretation. These limitations do not negate the clinical utility of plasma CGP, but they make performance context-dependent and highlight the need to define scenarios in which plasma testing is most informative (e.g., higher-ctF settings) versus when tissue testing or combined strategies are warranted [12,13,14].

Although several studies have investigated factors associated with the detection of therapeutic target genes using CGP, most of them include both tissue and liquid biopsies. There are a few reports that specifically examine factors associated with the detection of therapeutic target genes using liquid biopsy alone [15,16,17,18]. Therefore, we aimed to elucidate, in advanced BTC, the factors that influence the detection of actionable genomic alterations specifically through liquid biopsy.

## 2. Materials and Methods

### 2.1. Database of the Center for Cancer Genomics and Advanced Cancers (C-CAT)

This retrospective study used data from the C-CAT database [19,20]. This national database consolidates genomic and clinical data for research, and as of 2024, five CGP tests, including FoundationOne^®^ CDx [F1] (Foundation Medicine Inc., Cambridge, MA, USA) and OncoGuide™ NCC Oncopanel System [NOP] (Sysmex Co., Ltd., Kobe, Japan), FoundationOne^®^ Liquid CDx [F1L] (Foundation Medicine Inc., Cambridge, MA, USA), Guardant360^®^ CDx [G360] (Guardant Health Inc., Palo Alto, CA, USA) and GenMine TOP^®^ (Konica Minolta, Inc., Tokyo, Japan), have been approved for reimbursement by the Japanese public health insurance system.

From this database, we extracted data of patients who were diagnosed with BTC and included in the C-CAT database between June 2019 and January 2025. According to the OncoTree cancer classification platform, 7772 patients were classified as having biliary tract cancer, which was histologically confirmed. Among these patients, we excluded patients with TOP (n = 165) and G360 (n = 186) because, in the versions available during the study period (TOP 1.0.0 and Guardant360 GH2.11), MSI status could not be provided in the C-CAT database. Additionally, patients with NOP (n = 852) were also excluded and we restricted analyses to F1 (n = 5019) and F1L (n = 1550), which share a harmonized hybrid-capture design and vendor-maintained pipelines.

Data from the C-CAT database were downloaded on 16 January 2025, after approval by the Research Ethics Committee of Fukushima Medical University (registration number: REC2023-230) and the Information Utilization Review Board of C-CAT (CDU2024-022 N, 20 November 2024).

### 2.2. Genomic and Clinical Information of the Patients

Data on the following genomic alterations, which are recommended by the NCCN guidelines as therapeutic targets for molecular testing, were collected from the C-CAT database: MSI-H, TMB-H, *FGFR2*/*RET*/*NTRK* fusions, *BRAF V600E*, *KRAS G12C*, *IDH1* mutations, and *ERBB2* amplification. Clinical data were collected from the C-CAT database and included the following parameters: CGP test type, age, sex, cancer type (perihilar CCA [pCCA], and non-pCCA including iCCA, GBCa, and distal CCA), smoking and alcohol consumption history, presence of double cancer, family history of cancer, metastatic sites (liver, bone, lymph nodes/vessels, lung, and peritoneum), multiple metastases, multi-agent first-line chemotherapy, treatment line prior to CGP, and treatment response before CGP. Multi-agent first-line chemotherapy refers to cases where either GC (Gemcitabine + Cisplatin), GS (Gemcitabine + S-1), GCD (Gemcitabine + Cisplatin + Durvalumab), GCP (Gemcitabine + Cisplatin + Pembrolizumab), or GCS (Gemcitabine + Cisplatin + S-1) was administered. Because information was manually input into the database, some data were missing. Consequently, the percentages for each variable were calculated on the basis of the number of patients with available data, and the analyses were performed using data from patients with complete information.

### 2.3. Statistics

Categorical variables were summarized as counts and percentages and compared using the chi-square test. Variable-level missingness was tabulated by panel. To avoid listwise deletion and improve efficiency, covariate missingness was handled using multiple imputations (MI) by chained equations (m = 20; 50 iterations). Imputation models included the outcome and all analysis variables; analyses were performed separately in each imputed dataset and combined using Rubin’s rules. As a diagnostic, we compared baseline characteristics between complete-case and imputed F1L subsets using absolute standardized mean differences (SMDs).

For between-panel comparisons (F1L vs. F1), we estimated propensity scores via logistic regression including prespecified covariates available in both cohorts (age, dichotomized at the overall cohort median of 68 years [≥68 vs. <68]; sex; tumor category [non-pCCA]; ECOG-PS ≥ 2; smoking; alcohol polydipsia; double cancer; family history of cancer; metastatic sites [liver, bone, lymph node, lung, peritoneum]; treatment line at CGP ≥ 2; first-line regimen type; and disease status at CGP [progressive disease, PD]). One-to-one nearest-neighbor matching without replacement was performed with a caliper width equal to 0.2 times the standard deviation (SD) of the logit-transformed propensity score in the pre-matching sample. Covariate balance before and after matching was evaluated using absolute SMDs (target ≤ 0.10; ≤0.20 acceptable).

We modeled outcomes with logistic regression and reported odds ratios (ORs) with 95% confidence intervals in both the unmatched cohort (before matching) and the matched sample (after matching). In the matched analyses, we accounted for the pairing by using robust standard errors with each matched pair treated as a cluster. As an exploratory step, we also stratified results by how many prespecified enrichment factors each patient had (0–4). Two-sided *p* values < 0.05 were considered statistically significant. Analyses were performed in R (version 4.4.2) using tidyverse, janitor, naniar, mice, MatchIt, cobalt, gtsummary, broom, sandwich, and lmtest.

## 3. Results

### 3.1. Factors Associated with the Detection of Actionable Genomic Alterations in Liquid Biopsy

Within F1L cases, baseline characteristics were well balanced between the complete-case subset (n = 1001) and the imputed cohort (n = 1550), with all standardized mean differences < 0.20. Small imbalances included higher rates of smoking (SMD = 0.19), treatment line ≥ 2 at CGP (0.13), lymph-node metastasis (0.11), and family history of cancer (0.11) in the complete-case subset (Table 1).

In multivariable models, factors consistently associated with higher odds of detecting an actionable genomic alteration were non-pCCA (MI main: OR 2.05, 95% CI 1.33–3.15; CC main: OR 1.87, 1.13–3.24), liver metastasis (MI: 1.90, 1.43–2.52; CC: 1.89, 1.34–2.66), lymph node metastasis (MI: 1.41, 1.06–1.86; CC: 1.57, 1.12–2.22), and lung metastasis (MI: 1.52, 1.08–2.15; CC: 1.86, 1.23–2.79). Peritoneal dissemination showed a borderline inverse association (MI: OR 0.69, 0.47–1.01; *p* = 0.06). Age, sex, ECOG-PS, alcohol polydipsia, double cancer, family history, treatment line, and PD at CGP were not significant. In sensitivity models including treatment variables, combination chemotherapy was positively associated with MI (OR 1.63, 1.08–2.46; *p* = 0.02) but not in CC (*p* = 0.08) (Supplementary Table S1).

### 3.2. Detection Rate of Actionable Genomic Alterations After Propensity Score Matching

Overall missingness was low and comparable between panels (F1, n = 5019; F1L, n = 1550). Age, sex, and cancer type had no missing data. Most variables had ≤6% missingness—ECOG-PS 2.9–3.2%, smoking 5.6–6.3%, double cancer 2.8–3.4%, family history 4.7–5.3%—and all metastatic-site indicators were approximately 2% in both panels. The largest gaps were for first-line regimen (F1 17.5%, F1L 13.4%) and alcohol polydipsia (10.5–11.9%). Treatment line at CGP and PD at CGP each had 6–7% missingness (Table 2).

Before matching, baseline characteristics differed modestly between the F1L and F1 cohorts, most notably cancer type: non-pCCA was more frequent in F1 than F1L (88.5% vs. 78.6%; SMD = −0.27). Smaller imbalances included a higher use of combination first-line chemotherapy in F1L (73.2% vs. 68.0%; SMD = 0.11) and a greater proportion undergoing CGP at second line or later in F1 (49.3% vs. 43.6%; SMD = −0.11). After 1:1 matching (n = 1549 per group), covariates were well balanced, with all |SMD| ≤ 0.05 (including non-pCCA, SMD = −0.01) (Table 3).

In outcome comparisons, F1L consistently showed lower detection than F1 (Table 4). The proportion with any actionable genomic alteration was 16.8% with F1L versus 24.7% with F1 in the unmatched cohort (OR 0.61, 95% CI 0.53–0.71; *p* < 0.001), and this difference persisted after matching (16.8% vs. 24.8%; OR 0.61, 0.49–0.75; *p* < 0.001). TMB-high was likewise less frequent with F1L (unmatched: 4.4% vs. 6.7%; OR 0.64, 0.49–0.84; *p* = 0.001; matched: 4.4% vs. 6.9%; OR 0.62, 0.43–0.90; *p* = 0.01). *ERBB* amplification was markedly lower with F1L (unmatched: 4.6% vs. 10.6%; OR 0.40, 0.31–0.52; *p* < 0.001; matched: 4.6% vs. 10.3%; OR 0.42, 0.31–0.57; *p* < 0.001). No significant differences were observed for MSI-H, *IDH1*, *KRAS* G12C, or *BRAF* V600E after matching (all *p* > 0.05). *FGFR2* fusion/rearrangement was lower with F1L before matching (OR 0.61, 0.39–0.94; *p* = 0.02) and borderline after matching (OR 0.61, 0.34–1.08; *p* = 0.09). Estimates for very rare events (e.g., *NTRK*, *RET*) were imprecise.

### 3.3. Detection by Liquid Versus Tissue Across Strata of Enrichment-Factor Count

When stratified by the number of prespecified enrichment factors, the proportion of patients with an actionable genomic alteration detected by F1L increased from 5.8% with zero factors to 32.8% with four factors. In contrast, the F1 detection rate remained relatively stable, ranging between 23.6% and 25.1% across the same strata. Accordingly, the odds of detection with F1L versus F1 were substantially lower with zero factors (odds ratio [OR] 0.19, 95% confidence interval [CI] 0.08–0.44; *p* < 0.001) and one factor (OR 0.35, 0.22–0.55; *p* < 0.001), modestly lower with two factors (OR 0.68, 0.50–0.93; *p* = 0.020), indistinguishable with three factors (OR 1.04, 0.66–1.65; *p* = 0.870), and numerically higher but imprecise with four factors (OR 1.62, 0.48–5.39; *p* = 0.430). These findings indicate a dose–response enrichment for F1L, with performance approaching that of F1 at three or more factors (Table 5).

## 4. Discussion

In this study, four factors—non-perihilar tumor location and the presence of liver, lymph-node, or lung metastasis—were independently associated with higher detection of actionable genomic alterations on liquid CGP. Detection increased with the number of these factors, and patients with three or more showed rates comparable to tissue CGP. To our knowledge, this pattern has not been previously reported. These findings may aid patient selection for liquid biopsy in BTC.

Plasma ctDNA analysis for CGP, a form of liquid biopsy in cancer genomics, is a minimally invasive method that enables the detection of actionable genomic alterations from blood samples. In clinical practice, the utility of liquid biopsy is particularly relevant in BTC, where the success rate of tissue-based CGP is not always satisfactory [10,21]. For instance, a retrospective analysis by Zhang et al. reported a success rate of only 81.7% for CGP testing in BTC patients [22]. Similarly, Zill et al. demonstrated that in 26 BTC cases, 9 (35%) failed CGP due to insufficient tissue quantity or quality, resulting in an estimated success rate of approximately 65% [17]. These findings highlight the clinical need for liquid biopsy as a complementary approach, especially when tissue samples are inadequate or unavailable. However, negative liquid biopsy results may pose challenges in clinical decision-making. A lack of detectable actionable genomic alterations may either truly represent the tumor genotype (true negative) or indicate a false negative resulting from insufficient shedding of ctDNA, thereby missing an actionable oncogenic driver. To reduce the risk of false negatives, various approaches, including machine learning algorithms and advanced analytical techniques measuring the ctDNA tumor fraction, have been proposed to improve the accuracy of liquid CGP interpretation [18,23].

Although complex methodologies were not utilized in the present study, the present findings suggested that the detection rate of actionable genomic alterations may be estimated simply by counting the number of associated factors. The detection of actionable genomic alterations by liquid biopsy is determined by the characteristics of the tumor itself (tumor type, histological type and location) as well as the quantity of ctDNA [24,25,26]. Additionally, it is well known that the amount of ctDNA is greater in patients with liver metastasis, lung metastasis, lymph node metastasis, or peritoneal dissemination than in those with other metastatic sites [27,28].

Previous studies have reported that genomic expression differs depending on the lesion site, such as higher detection rates of *FGFR2* fusion genes and *IDH1* mutations in iCCA [29,30]. Similar findings have also been reported for liquid CGP [16]. The selected factors in the present study were appropriate, as they are each related to the detection of therapeutically relevant genomic alterations. The novelty of the present study lies in the focus on the association between the number of these factors and the detection rate of actionable genomic alterations in liquid biopsy.

Tissue CGP plausibly yields higher detection rates for TMB-high and *ERBB2* amplification because plasma assays are intrinsically constrained by circulating tumor fraction (ctF) and by the physics of cfDNA. FoundationOne Liquid CDx (F1L), for example, requires relatively high ctF to confidently call copy-number gains; its FDA technical documentation indicates that the median limit of detection for copy-number amplifications corresponds to approximately 20–25% ctF (*ERBB2*: 19.8%). Consequently, true *ERBB2* amplifications may go uncalled when ctF is lower—a common circumstance in biliary tract cancers—whereas tissue CGP, which is not limited by ctF, typically provides deeper and more uniform coverage and a broader dynamic range for copy-number inference in FFPE, improving sensitivity for *ERBB2* amplification.

For TMB, plasma-based estimates are more susceptible to dilution and sampling noise because they depend on the number of tumor-derived DNA molecules captured from circulation. Low ctF reduces the effective callable territory and variant count, biasing TMB downward and making TMB-high status harder to confirm. By contrast, tissue assays such as F1 analyze higher tumor DNA input with consistent hybrid-capture coverage and validated pipelines, producing more stable mutation counts across a large exonic footprint and, therefore, more reliable TMB classification. Together, these analytical realities—ctF dependence, reduced copy-number dynamic range in cfDNA, and greater stochastic variance in variant detection—provide a coherent explanation for the higher rates of TMB-high and *ERBB2* amplification observed with tissue CGP relative to plasma CGP, even when analyses are restricted to closely harmonized panels (F1 vs. F1L).

The present study had several limitations. First, nearly half of the patients (4463 out of 7772) were excluded from the analysis because of missing data, which may have introduced potential selection bias. Second, because this study was not designed with matched tissue–plasma CGP from the same patients, false negatives in plasma cannot be excluded and inter-patient heterogeneity may have affected detection rates and predictor performance. Thus, direct comparisons between tissue- and liquid-based CGP should ideally be performed in paired analyses. Our findings are therefore hypothesis-generating and warrant validation in large cohorts with paired tissue and liquid CGP.

## 5. Conclusions

The utility of liquid CGP can be maximized by selecting patients in whom detection is likely to be higher. In our cohort, detection was enriched in those with non-perihilar tumors and with liver, lymph-node, or lung metastases, and rates approached those of tissue CGP when three or more of these factors were present. Although liquid CGP generally yields lower overall detection than tissue CGP, triaging to these clinical contexts can increase the likelihood of identifying actionable alterations in BTC.

## Figures and Tables

**Table 1:** Baseline characteristics of F1L cases: complete-case subset vs. imputed cohort.

	Complete Case (n = 1001)	Imputed Cases (n = 1550)	SMD
Age > 68 years old, n (%)	56.9	61.6	−0.09
Gender, male, n (%)	59.3	63.4	−0.08
Cancer type, Non-pCCA, n (%)	78.1	79.6	−0.04
ECOG-PS > 2, n (%)	3.0	3.1	−0.02
Smoking, yes, n (%)	57.2	48.0	0.19
Alcohol polydipsia, n (%)	17.9	16.1	0.05
Double cancer, n (%)	11.4	10.5	0.03
Family history of cancer, n (%)	71.5	66.6	0.11
Liver metastasis, n (%)	36.4	37.0	−0.01
Bone metastasis, n (%)	7.2	6.7	0.02
Lymph nodes metastasis, n (%)	47.2	41.8	0.11
Lung metastasis, n (%)	17.1	19	−0.05
Peritoneum dissemination, n (%)	20	19.3	0.02
1st line chemotherapy regimen, combination, n (%)	79.7	78	0.04
Treatment line at CGP registration, 2nd or later, n (%)	45.3	38.9	0.13
Chemotherapy response at CGP, PD, n (%)	17.4	17.1	0.01

SMD, standardized mean difference; pCCA, perihilar cholangiocarcinoma; ECOG-PS, Eastern Cooperative Oncology Group performance status; CGP, comprehensive genomic profiling; PD, progression disease. Data are shown as %.

**Table 2:** Missingness per panel.

	F1 (n = 5019)	F1L (n = 1550)
Age > 68 years old, n (%)	0 (0.0)	0 (0.0)
Gender, male, n (%)	0 (0.0)	0 (0.0)
Cancer type, non-pCCA, n (%)	0 (0.0)	0 (0.0)
ECOG-PS > 2, n (%)	147 (2.9)	50 (3.2)
Smoking, yes, n (%)	282 (5.6)	97 (6.3)
Alcohol polydipsia, n (%)	530 (10.5)	184 (11.9)
Double cancer, n (%)	171 (3.4)	43 (2.8)
Family history of cancer, n (%)	264 (5.3)	73 (4.7)
Liver metastasis, n (%)	107 (2.1)	31 (2.0)
Bone metastasis, n (%)	107 (2.1)	31 (2.0)
Lymph nodes metastasis, n (%)	107 (2.1)	31 (2.0)
Lung metastasis, n (%)	107 (2.1)	31 (2.0)
Peritoneum dissemination, n (%)	107 (2.1)	31 (2.0)
1st line chemotherapy regimen, combination, n (%)	877 (17.5)	208 (13.4)
Treatment line at CGP registration, 2nd or later, n (%)	351 (6.9)	99 (6.3)
Chemotherapy response at CGP, PD, n (%)	365 (7.3)	105 (6.7)

SMD, standardized mean difference; pCCA, perihilar cholangiocarcinoma; ECOG-PS, Eastern Cooperative Oncology Group performance status; cStage, clinical stage; Met, metastasis; CGP, comprehensive genomic profiling; PD, progression disease. Data are shown as n (%).

**Table 3:** Baseline characteristics of F1L versus F1 before and after propensity-score matching (unmatched and matched cohorts).

	Unmatched			Matched		
	F1L (n = 1550)	F1 (n = 5019)	SMD	F1L (n = 1549)	F1L (n = 1549)	SMD
Age > 68 years old	58.6	55.4	0.06	58.6	57.5	0.02
Gender, male	60.8	61.7	−0.02	60.8	60.7	0.00
Cancer type, non-pCCA	78.6	88.5	−0.27	78.7	78.9	−0.01
ECOG-PS > 2	3.0	2.4	0.04	3.0	2.5	0.03
Smoking	54.4	53.0	0.03	54.3	54.2	0.00
Alcohol polydipsia	17.3	16.6	0.02	17.3	16.6	0.02
Double cancer	11.1	12.6	−0.05	11.1	9.5	0.05
Family history of cancer	70.0	71.2	−0.03	70.0	70.0	0.00
Liver metastasis	36.5	38.9	−0.05	36.6	36.0	0.01
Bone metastasis	7.0	8.5	−0.05	7.0	6.1	0.04
Lymph nodes metastasis	45.3	45.6	−0.01	45.3	45.5	0.00
Lung metastasis	17.4	19.4	−0.05	17.4	16.2	0.03
Peritoneum dissemination	19.8	20.3	−0.01	19.7	19.1	0.02
1st line chemotherapy regimen, combination	73.2	68.0	0.11	73.2	73.9	−0.01
Treatment line at CGP registration, 2nd or later	43.6	49.3	−0.11	43.7	43.8	0.00
Chemotherapy response at CGP, PD	17.4	18.5	−0.03	17.4	16.0	0.04

F1L, FoundationOne^®^ Liquid CDx; F1, FoundationOne^®^ CDx; SMD, standardized mean difference; pCCA, perihilar cholangiocarcinoma; ECOG-PS, Eastern Cooperative Oncology Group performance status; CGP, comprehensive genomic profiling; PD, progression disease. Data are shown as n (%). Data are shown as mean (%).

**Table 4:** Detection rate of actionable genomic alterations before and after propensity-score matching (unmatched and matched cohorts).

	Unmatched				Matched			
Outcome	F1L (%)	F1 (%)	OR (95% CI)	*p*	F1L (%)	F1 (%)	OR (95% CI)	*p*
Actionable genomic alterations	16.8	24.7	0.61 (0.53–0.71)	<0.001	16.8	24.8	0.61 (0.49–0.75)	<0.001
TMB-H	4.4	6.7	0.64 (0.49–0.84)	0.001	4.4	6.9	0.62 (0.43–0.90)	0.01
MSI-H	1.5	2.2	0.69 (0.44–1.09)	0.11	1.5	2.4	0.66 (0.35–1.21)	0.17
*ERBB* amplification	4.6	10.6	0.40 (0.31–0.52)	<0.001	4.6	10.3	0.42 (0.31–0.57)	<0.001
*BRAF* V600E	0.5	0.7	0.74 (0.34–1.59)	0.44	0.5	0.7	0.74 (0.26–2.08)	0.56
*IDH1*	5.5	5.5	1.01 (0.78–1.29)	0.96	5.6	5.7	0.97 (0.69–1.36)	0.86
*KRAS* G12C	1.2	1.3	0.91 (0.54–1.54)	0.72	1.2	1.4	0.81 (0.41–1.59)	0.54
*FGFR2* fusion or rearrangement	1.6	2.6	0.61 (0.39–0.94)	0.02	1.6	2.6	0.61 (0.34–1.08)	0.09
*NTRK* fusion	0.2	0.1	1.95 (0.46–8.15)	0.36	0.2	0.1	21.1 (95% CI: NE)	0.99
*RET* fusion	0	0	0 (95% CI: NE)	0.99	0	0	0 (95% CI: NE)	1.00

F1L, FoundationOne^®^ Liquid CDx; F1, FoundationOne^®^ CDx; OR, odds ratio; CI, confidential interval; TMB, tumor mutational burden; MSI, microsatellite instability; *ERBB2*, erb-b2 receptor tyrosine kinase 2; *BRAF*, v-Raf murine sarcoma viral oncogene homolog B; *KRAS*, Kirsten rat sarcoma viral oncogene homolog; *IDH*, isocitrate dehydrogenase; *FGFR*, fibroblast growth factor receptor; *NTRK*, neurotrophic tyrosine receptor kinase; *RET*, rearranged during transfection.

**Table 5:** Detection rate of actionable genomic alterations by number of enrichment factors in propensity-score-matched cohort.

Number of Items	F1L (%)	F1 (%)	OR	*p*
0	5.8	24.2	0.19 (0.08–0.44)	<0.001
1	10.5	25.1	0.35 (0.22–0.55)	<0.001
2	18.5	24.9	0.68 (0.50–0.93)	0.020
3	25.2	24.6	1.04 (0.66–1.65)	0.870
4	32.8	23.6	1.62 (0.48–5.39)	0.430

F1L, FoundationOne^®^ Liquid CDx; F1, FoundationOne^®^ CDx; OR, odds ratio; CI, confidential interval.

## Data Availability

The data analyzed in this study are available from C-CAT (https://www.ncc.go.jp/en/c_cat/about/index.html (accessed on 16 January 2025)). Access to these data was restricted but was granted for this study. Data are available from the authors upon reasonable request with permission from the C-CAT.

## Supplementary Materials

The following supporting information can be downloaded at: https://www.mdpi.com/article/10.3390/cancers17183071/s1, Table S1: Multivariable logistic regression of factors associated with detection of actionable genomic alterations in F1L: complete-case and multiple-imputation models.
