# Supplementary material for: Factors Associated with the Detection of Actionable Genomic Alterations Using Liquid Biopsy in Biliary Tract Cancer
Source: Cancers (Basel). 2025 Sep 19;17(18):3071. doi: 10.3390/cancers17183071 (PMC12468581; doi:10.3390/cancers17183071)
Supplement: Supplementary file 1 [file cancers-17-03071-s001.zip › cancers-3846601-supplementary.pdf]

Supplementary Table S1. Multivariable logistic regression of factors associated with detection of actionable genomic alterations in F1L: complete-case and multiple-imputation models.

Model	Variable	OR (95% CI)	P
CC main (no treatment)	Age>68 years-old	0.82 (0.58-1.16)	0.26
CC main (no treatment)	Gender, male	0.96 (0.63-1.46)	0.83
CC main (no treatment)	Cancer type, non-pCCA	1.87 (1.13-3.24)	0.02
CC main (no treatment)	Smoking	0.95 (0.62-1.45)	0.80
CC main (no treatment)	Alcohol polydipsia	1.17 (0.73-1.83)	0.52
CC main (no treatment)	Double cancer	0.79 (0.43-1.38)	0.43
CC main (no treatment)	Family history of cancer	1.30 (0.88-1.94)	0.19
CC main (no treatment)	Liver metastasis	1.89 (1.34-2.66)	<0.01
CC main (no treatment)	Bone metastasis	1.29 (0.70-2.28)	0.40
CC main (no treatment)	Lymph nodes metastasis	1.57 (1.12-2.22)	0.01
CC main (no treatment)	Lung metastasis	1.86 (1.23-2.79)	<0.01
CC main (no treatment)	Peritoneum dissemination	0.67 (0.41-1.06)	0.10
CC sensitivity (+treatment)	Age>68 years-old	0.84 (0.60-1.19)	0.33
CC sensitivity (+treatment)	Gender, male	0.99 (0.65-1.52)	0.97
CC sensitivity (+treatment)	Cancer type, non-pCCA	1.90 (1.15-3.30)	0.02
CC sensitivity (+treatment)	Smoking	0.92 (0.60-1.41)	0.69
CC sensitivity (+treatment)	Alcohol polydipsia	1.16 (0.73-1.83)	0.52
CC sensitivity (+treatment)	Double cancer	0.81 (0.44-1.41)	0.47
CC sensitivity (+treatment)	Family history of cancer	1.28 (0.87-1.92)	0.21
CC sensitivity (+treatment)	Liver metastasis	1.94 (1.38-2.75)	<0.01
CC sensitivity (+treatment)	Bone metastasis	1.26 (0.68-2.24)	0.44
CC sensitivity (+treatment)	Lymph nodes metastasis	1.60 (1.13-2.27)	<0.01
CC sensitivity (+treatment)	Lung metastasis	1.84 (1.21-2.77)	<0.01
CC sensitivity (+treatment)	Peritoneum dissemination	0.68 (0.41-1.07)	0.10
CC sensitivity (+treatment)	1st line chemotherapy regimen, combination	1.50 (0.97-2.41)	0.08
CC sensitivity (+treatment)	Treatment line at CGP registration, 2nd or later	1.16 (0.82-1.63)	0.40
CC sensitivity (+treatment)	Chemotherapy response at CGP, PD	0.78 (0.49-1.22)	0.29
MI main (no treatment)	Age>68 years-old	0.79 (0.60-1.05)	0.10
MI main (no treatment)	Gender, male	1.12 (0.80-1.57)	0.51
MI main (no treatment)	Cancer type, non-pCCA	2.05 (1.33-3.15)	<0.01
MI main (no treatment)	ECOG-PS>2	1.04 (0.78-1.39)	0.79
MI main (no treatment)	Smoking	0.71 (0.50-1.01)	0.06

MI main (no treatment)	Alcohol polydipsia	1.14 (0.76-1.69)	0.53
MI main (no treatment)	Double cancer	1.00 (0.63-1.57)	1.00
MI main (no treatment)	Family history of cancer	1.27 (0.92-1.74)	0.15
MI main (no treatment)	Liver metastasis	1.90 (1.43-2.52)	<0.01
MI main (no treatment)	Bone metastasis	1.38 (0.85-2.25)	0.19
MI main (no treatment)	Lymph nodes metastasis	1.41 (1.06-1.86)	0.02
MI main (no treatment)	Lung metastasis	1.52 (1.08-2.15)	0.02
MI main (no treatment)	Peritoneum dissemination	0.69 (0.47-1.01)	0.06
MI sensitivity (+treatment)	Age>68 years-old	0.82 (0.62-1.09)	0.17
MI sensitivity (+treatment)	Gender, male	1.15 (0.81-1.61)	0.43
MI sensitivity (+treatment)	Cancer type, non-pCCA	2.07 (1.34-3.19)	<0.01
MI sensitivity (+treatment)	ECOG-PS>2	1.07 (0.80-1.43)	0.65
MI sensitivity (+treatment)	Smoking	0.70 (0.49-0.99)	0.04
MI sensitivity (+treatment)	Alcohol polydipsia	1.15 (0.77-1.71)	0.49
MI sensitivity (+treatment)	Double cancer	1.02 (0.65-1.62)	0.92
MI sensitivity (+treatment)	Family history of cancer	1.25 (0.91-1.73)	0.17
MI sensitivity (+treatment)	Liver metastasis	1.91 (1.44-2.54)	<0.01
MI sensitivity (+treatment)	Bone metastasis	1.39 (0.85-2.25)	0.19
MI sensitivity (+treatment)	Lymph nodes metastasis	1.40 (1.06-1.86)	0.02
MI sensitivity (+treatment)	Lung metastasis	1.48 (1.05-2.11)	0.03
MI sensitivity (+treatment)	Peritoneum dissemination	0.68 (0.46-1.00)	0.05
MI sensitivity (+treatment)	1st line chemotherapy regimen, combination	1.63 (1.08-2.46)	0.02
MI sensitivity (+treatment)	Treatment line at CGP registration, 2nd or later	1.06 (0.80-1.43)	0.67
MI sensitivity (+treatment)	Chemotherapy response at CGP, PD	1.02 (0.70-1.48)	0.92

CC, complete case; MI, multiple imputation; OR, odds ratio; CI, confidential interval; SMD, standardized mean difference; pCCA, perihilar cholangiocarcinoma; ECOG PS, Eastern Cooperative Oncology Group performance status; CGP, comprehensive genomic profiling; PD, progression disease. Data are shown as %.
